# Supplementary material for: Rapid turnover of life-cycle-related genes in the brown algae
Source: Genome Biol. 2019 Feb 14;20:35. doi: 10.1186/s13059-019-1630-6 (PMC6374913; doi:10.1186/s13059-019-1630-6)
Supplement: Supplementary file 3 — Protein alignments, corresponding Gblocks html file and CDS sequences for the lineage conserved gametophyte-biased genes. (ZIP 2109 kb) [file 13059_2019_1630_MOESM3_ESM.zip › Con_GA_alignments/Ec-01_000080.aa_ali.fasta-gb.htm]

Ec-01\_000080.aa.fasta.trans.aa\_ali.fasta


## Gblocks 0.91b Results

Processed file: **Ec-01\_000080.aa.fasta.trans.aa\_ali.fasta**  
Number of sequences: **2**  
Alignment assumed to be: **Protein**  
New number of positions: **553** (selected positions are underlined in blue)

```
                         10        20        30        40        50        60
                 =========+=========+=========+=========+=========+=========+
Ec-01_000080     --------------MASQSSSNPNQLLPFEQAWRRSGQDYFDRFPQENGSIFLFNTYTGE
MSTRG.53315.1    ARRGARRPCVLYRTMSCEGTYPSGELLPYCKAWKRSGLVFFEAFPQANGSTFLFNTYTGE
                                          ###################################


                         70        80        90       100       110       120
                 =========+=========+=========+=========+=========+=========+
Ec-01_000080     AIYDVDRWGSPLPGARTLSHWFVPTAVVPP-ATEGEPVDTHNLRDLRDDFPGSEYRARWH
MSTRG.53315.1    AIYDVDRWGSPLPDARKLSHWFEPTAEVPPDGDEDDLLRPRKLRDLRGDFPGNEYRARWH
                 ##############################   ###########################


                        130       140       150       160       170       180
                 =========+=========+=========+=========+=========+=========+
Ec-01_000080     CAELIQRNVRAALSRWRVARALCAVWVKDYDHEAERFSFTHQNNREMQYHKPWGMGTVDL
MSTRG.53315.1    CAEVIQRNARAALSRWKIARALWSTWVKKYDTEAERYSFTHKATGQMQYHKPWGMGTVDL
                 ############################################################


                        190       200       210       220       230       240
                 =========+=========+=========+=========+=========+=========+
Ec-01_000080     WAVPGEKVDEHLVSLFLRVESATAEERTDEEPRDWEASKRHRRRLYKFYQEKKFSEGPHC
MSTRG.53315.1    WAVPGDKADKNLASLFLGVKSVASQDNADDEPRDWEASRRYRRRLYDFYQEKNFSEGPHC
                 ############################################################


                        250       260       270       280       290       300
                 =========+=========+=========+=========+=========+=========+
Ec-01_000080     RIVGPGSLKESSFTNVFLYPKKYYKTKPFSHPRDRQCEGARMGSQVTCMDGLVEKRISAD
MSTRG.53315.1    RIVGPGSLKETSFTNVFLHPKKYHKTRPFAHPRDRQFEGARIGTYVKCMDHLVEKRIRAD
                 ############################################################


                        310       320       330       340       350       360
                 =========+=========+=========+=========+=========+=========+
Ec-01_000080     AYFHVRNASEHGALNVIKLMFKHRDDLRVQGVGLQRMAGSLLEEDEAGGATEGQKKYLEK
MSTRG.53315.1    AYFHVRNASEHGALNVLKVMFKYRDNVHVQGIGLQRMAGSLLEEDEAGGATEGQKKYLEE
                 ############################################################


                        370       380       390       400       410       420
                 =========+=========+=========+=========+=========+=========+
Ec-01_000080     AFQLLRETPNVEWTQAEACAALASMTSGLCIRKEIDREHADWLEDVVTAIMGVKSITQTV
MSTRG.53315.1    SLELLRETPNIEWIQAEACAALASMTSGLCNRKEIDRQHPDWLEDVVTAIMGVKTITQTV
                 ############################################################


                        430       440       450       460       470       480
                 =========+=========+=========+=========+=========+=========+
Ec-01_000080     KEIDPDGKETRYDVRVPSPMAHLVALHGCRILANMACDDHNRAEVARGSIGAVVHVANLM
MSTRG.53315.1    KQIDPDGTETRYDVTIPSPMAHLIALHGCRILANMACDEHNRAQVARGSIAAVVHVAKLM
                 ############################################################


                        490       500       510       520       530       540
                 =========+=========+=========+=========+=========+=========+
Ec-01_000080     ADDPVLVCAVSAAVYNFVSRQETTHKIVVEAGTAEALEGMLKNLEYTDRKEKELVTRALT
MSTRG.53315.1    ADDPVLACTISATVYNFVSRQELTHQIVVEAGTVEALEGMQSNLERTDLREKEMVVRALT
                 ############################################################


                        550       560       570       580       590       600
                 =========+=========+=========+=========+=========+=========+
Ec-01_000080     ALEPDGWRGVDHGLRDEDIPEHVDESSTSVDDSISGMGLTIASNNAGEGTVSAMVSPADG
MSTRG.53315.1    ALEPEGWRGVDHGLKDEDIPQD-DESSTSLDGSSG-----I-------------VSSTAS
                 ####################   ##########                     ######


                 
                 ======
Ec-01_000080     CEKNAT
MSTRG.53315.1    QLKTAR
                 #####
```

```
Parameters used
Minimum Number Of Sequences For A Conserved Position: 2
Minimum Number Of Sequences For A Flanking Position: 2
Maximum Number Of Contiguous Nonconserved Positions: 8
Minimum Length Of A Block: 10
Allowed Gap Positions: None
Use Similarity Matrices: Yes
```

```
Flank positions of the 4 selected block(s)
Flanks: [26  90]  [94  560]  [564  573]  [595  605]  

New number of positions in Ec-01_000080.aa.fasta.trans.aa_ali.fasta-gb:  553  (91% of the original 606 positions)
```
